# Fructose and metabolic health: governed by hepatic glycogen status?

**DOI:** 10.1113/JP277767

**Published:** 2019-04-21

**Authors:** Aaron Hengist, Francoise Koumanov, Javier T. Gonzalez

**Affiliations:** ^1^ Department for Health University of Bath Bath UK

**Keywords:** fructose, liver, hepatic, glycogen, de novo lipogenesis, metabolism

## Abstract

Fructose is a commonly ingested dietary sugar which has been implicated in playing a particularly harmful role in the development of metabolic disease. Fructose is primarily metabolised by the liver in humans, and increases rates of hepatic *de novo* lipogenesis. Fructose increases hepatic *de novo* lipogenesis via numerous mechanisms: by altering transcriptional and allosteric regulation, interfering with cellular energy sensing, and disrupting the balance between lipid synthesis and lipid oxidation. Hepatic *de novo* lipogenesis is also upregulated by the inability to synthesise glycogen, either when storage is inhibited in knock‐down animal models or storage is saturated in glycogen storage disease. Considering that fructose has the capacity to upregulate hepatic glycogen storage, and replenish these stores more readily following glycogen depleting exercise, the idea that hepatic glycogen storage and hepatic *de novo* lipogenesis are linked is an attractive prospect. We propose that hepatic glycogen stores may be a key factor in determining the metabolic responses to fructose ingestion, and saturation of hepatic glycogen stores could exacerbate the negative metabolic effects of excessive fructose intake. Since physical activity potently modulates glycogen metabolism, this provides a rationale for considering nutrient–physical activity interactions in metabolic health.

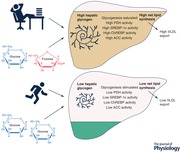

## Introduction

Fructose is a hexose with an identical chemical formula to glucose (C_6_H_12_O_6_), but with a keto group in position two of its carbon chain instead of an aldehyde group in position one of the carbon chain (Tappy & Lê, 2010). Whilst some fructose can be endogenously produced (Hwang *et al*. 2017), most fructose becomes available to humans from the diet. In Europe, it is estimated that two‐thirds of dietary fructose is consumed as sucrose (a glucose–fructose disaccharide, commonly known as ‘table sugar’) and around one‐third is ingested as free fructose (Sluik *et al*. 2015), although even when fructose is consumed in the free form, it is rarely consumed without the co‐ingestion of glucose (either as free glucose or glucose polymers). Common food sources of fructose intake in Europe include soft drinks (sugar sweetened beverages), fruit juices, fruits, cakes and dairy products (Sluik *et al*. 2015). Reported intake of free sugars, including fructose, is quantitatively important, ranging from ∼40 to 100 g per day across developed nations (roughly 7–20% of total energy intake) (Wittekind & Walton, 2014). The role of fructose in the human diet could be viewed as contentious, since some would argue that fructose is a uniquely harmful sugar for metabolic health and should be essentially avoided by all (Lustig *et al*. 2012), whereas others would argue that certain populations have exquisite metabolic health in the presence of extremely high fructose intakes (Pontzer *et al*. 2018), and there are even some recommendations for fructose‐containing carbohydrates to optimise performance and recovery during competition and intensive training in athletes (Gonzalez *et al*. 2016, 2017). In an attempt to solve this conflict on the role(s) of dietary fructose, this symposium review will aim to unify the related symposium reviews (Fuchs *et al*. 2019; Pinnick & Hodson, 2019; Tappy & Rosset, 2019; von Holstein‐Rathlou, 2019) by demonstrating how moderating physiological factors are important to consider when assessing the impact of fructose ingestion on metabolic health. Since fructose is a key contributor to disorders of fat metabolism, the role of fructose in hepatic lipogenesis will be a key focus. We will present the hypothesis that hepatic glycogen stores may regulate metabolic responses to fructose ingestion and could therefore be a target to prevent or mitigate the negative metabolic effects of fructose intake.

## Dietary carbohydrate intake

Dietary carbohydrates reportedly comprise ∼46% (224 g per day) of energy intake in the UK, with ‘free sugars’ comprising ∼11% (57 g per day) (Roberts *et al*. 2018). Dietary carbohydrates are commonly classified as mono‐/disaccharides composed of one/two monomers (e.g. glucose, fructose, galactose), oligosaccharides composed of typically 3–10 monomers (e.g. maltodextrins, raffinose), or polysaccharides composed of many monomers (e.g. amylose, amylopectin) (Scientific Advisory Committee on Nutrition, 2015). With regards to hepatic metabolism of carbohydrate, ingestion of glucose in either free form, or as the various polymers such as maltose, maltodextrin and (amylose) starch can be considered physiologically similar stimuli because hydrolysis of glucose polymers is not thought to be rate‐limiting to intestinal absorption (Gonzalez *et al*. 2017). Furthermore, free glucose is rarely ingested alone as a sugar and, for this reason, it has been proposed that the ingestion of glucose alone is more reflective of non‐sugar intake from a physiological perspective (Tappy, 2018). In other words, when referring to sugar intake, we are typically referring to the co‐ingestion of fructose and glucose, and not the ingestion of free glucose alone. With this in mind, public health strategies that aim to reduce the intake of free sugars (Scientific Advisory Committee on Nutrition, 2015), such as the Soft Drinks Industry Levy in the UK (Barber, 2017) will, if successful, essentially reduce the intakes of fructose‐containing carbohydrates.

## Metabolic health and postprandial lipid metabolism

Metabolic health is an umbrella term which can be defined as the ability to maintain homeostasis of substrates in response to challenging stimuli (including exercise and nutrition). It can be inferred at many levels, from molecular to whole body. Metabolic health is often characterised as the ability to maintain blood glucose or lipid concentrations within a range that does not increase risk of disease (Edinburgh *et al*. 2017). This is important for cardiovascular disease, for example, because fasting and postprandial hyperglycaemia and hyperlipaemia are associated with the development of cardiovascular disease (Edinburgh *et al*. 2017). Fructose intake has been implicated to play a role in many facets of metabolic health (Tappy & Lê, 2010) but, due to the notion that fructose is a key contributor towards disorders of fat metabolism (Softic *et al*. 2016), this review will focus on lipid handling.

In healthy humans, in the overnight fasted state, circulating blood lipid concentrations are composed of non‐esterified fatty acids (NEFAs) and triacylglycerols. When humans ingest a meal containing fat, exogenous fat is digested and broken down into NEFAs and monoglycerides before undergoing re‐esterification in the intestine and appearing in the circulation packaged in chylomicrons. Blood triacylglycerol concentrations increase steadily following ingestion of a meal and peak ∼5 h following ingestion (Chavez‐Jauregui *et al*. 2010), whereas NEFA concentrations typically decrease to negligible circulating concentrations within the first hour of ingestion, especially if the meal elicits an insulin response (Vega‐Lopez *et al*. 2013). Adipose tissue, skeletal muscle and the liver function integratively to manage postprandial lipaemia and most lipids are stored as triglycerides in adipose tissue (Frayn *et al*. 2006). The usual site of lipid storage is the adipose tissue, whereas lipid handling in non‐adipose tissues – including the liver – can cause a burden to these organs.

Compared to glucose ingestion, fructose ingestion (at a dose of 0.75 g⋅kg body mass^−1^) can increase the postprandial lipaemic response to the first meal of the day in healthy individuals (Abraha *et al*. 1998). This is predominantly due to a lower insulinaemic response following fructose ingestion compared with glucose ingestion, whereby lipoprotein lipase (LPL) activity in adipose tissue is activated less and clearance of dietary triacylglyceride (TAG) into adipose tissue is reduced (Chong *et al*. 2007). This dose of fructose equates to 52.5 g of fructose for a 70 kg individual; for context, a 330 ml can of Coca‐Cola contains ∼35 g of sucrose, so this is a high dose of fructose which would not usually be consumed in bolus outside the laboratory. Meta‐analysis of studies where the diet has been supplemented with fructose shows an elevation of postprandial lipaemia only in individuals with overweight or obesity and not in otherwise healthy participants (Wang *et al*. 2014). This appears to be driven by a disproportional increase in *de novo* lipogenesis (DNL) and liver fat accumulation with more prolonged (i.e. 3 weeks) fructose overfeeding (Sevastianova *et al*. 2012), and liver fat correlates strongly with hepatic very‐low‐density lipoprotein (VLDL) production and serum TAG concentrations (Adiels *et al*. 2006). Thus, the insulin response appears to be the main factor dictating acute responses to fructose ingestion, but longer‐term detriments are characterised by increased hepatic lipogenesis. This suggests that dietary fructose ingestion may be particularly harmful for health in certain contexts, for example when humans are in a positive energy balance and/or low energy turnover.

## Hepatic lipid and carbohydrate handling, hepatic *de novo* lipogenesis, and interactions with fructose

The mechanisms that drive the differences in fructose‐induced hypertriglyceridaemia under various levels of energy balance and energy turnover are likely to be mediated by hepatic metabolism of fructose and lipids. Whilst the intestine can metabolise some fructose (Jang *et al*. 2018), the liver is the primary site of fructose metabolism in humans (Tappy & Lê, 2010; Gonzalez & Betts, 2018). In the postabsorptive state, NEFA is the primary substrate for hepatic fat oxidation and acts as a precursor for TAG synthesis (Havel *et al*. 1970). The liver contributes to handling postprandial lipaemia by taking up remnant lipoprotein particles driven by the enzyme hepatic lipase. The liver also has capacity for DNL (Sanders & Griffin, 2016), which is the formation of new lipids from non‐lipid precursors. Therefore, hepatic lipid content is dependent on the rates of hepatic fatty acid uptake and synthesis on the one hand, and rates of fatty acid oxidation and secretion (as VLDL) on the other. Excessive lipid accumulation in the liver or in the circulation are each thought to be detrimental to health. Thus, notwithstanding the intrinsic links between hepatic fatty acid oxidation, DNL and VLDL secretion (Nguyen *et al*. 2008), perhaps the ultimate fate of net lipid synthesis (VLDL secretion or hepatic TAG storage) is less important than the processes of hepatic lipid uptake, synthesis, and oxidation. In other words, the mechanisms of hepatic lipid uptake, synthesis, and oxidation are likely the most important targets to regulate metabolic health in relation to hepatic lipid metabolism, as the downstream partitioning to VLDL secretion or hepatic TAG storage are both detrimental to metabolic health when excessively stimulated.

**Figure 1:**
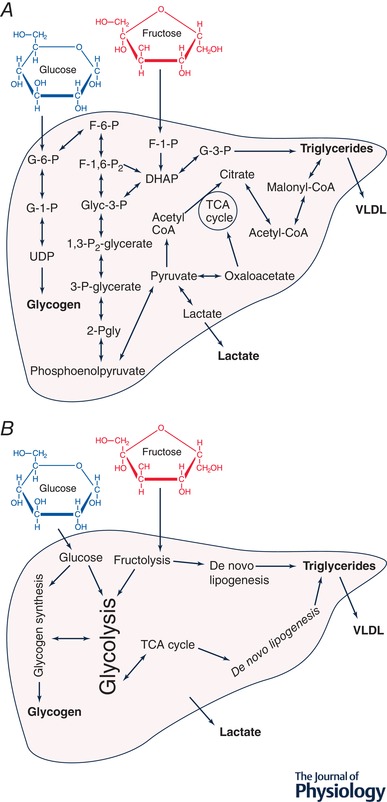
Pathways of glucose and fructose metabolism in the liver *A*, major pathways of hepatic glycogen synthesis and *de novo* lipogenesis. Hepatic glucose uptake is tightly regulated, whereas hepatic fructose uptake occurs in an ‘unregulated’ manner, without negative feedback, driven by the highly affinitive hepatic fructokinase. DNL can occur from glucose and fructose as precursors via multiple pathways: either via increased glycolytic production of acetyl coenzyme A, or via accumulation of dihydroxyacetone phosphate from glycolytic (fructose‐1,6‐bisphosphate) or directly from fructolytic (fructose‐1‐phosphate) intermediates. *B*, the major processes involved in hepatic glycogen synthesis and *de novo* lipogenesis which summarise the multiple enzymatic steps highlighted in *A*. G‐6‐P, glucose‐6‐phosphate; G‐1‐P, glucose‐1‐phosphate; UDP, uridine diphosphate glucose; F‐1‐P, fructose‐1‐phosphate; F‐6‐P, fructose‐6‐phosphate; F‐1,6‐P_2_, fructose‐1,6‐bisphosphate; Glyc‐3‐P, glyceraldehyde‐3‐phosphate; 1,3‐P_2_‐glycerate, 1,3‐bisphosphoglycerate; G‐3‐P, glycerol‐3‐phosphate; 3‐Pgly, 3‐phosphoglycerate; 2‐Pgly, 2‐phosphoglycerate; Acetyl‐CoA, acetyl coenzyme A; Malonyl‐CoA, malonyl coenzyme A; DHAP, dihydroxyacetone phosphate; VLDL, very‐low‐density lipoprotein.

At rest, the majority of the fructose (when ingested alone) taken up by the liver contributes to gluconeogenesis and some is converted to liver glycogen (Tappy & Lê, 2010). However, fructose also influences how the liver responds to glucose; infusing fructose into the portal vein of dogs in a stepwise manner results in an incremental rise in net hepatic glucose uptake (Shiota *et al*. 1998), and in humans, fructose infusion markedly upregulates hepatic glycogen synthesis (Petersen *et al*. 2001). Some fructose will be metabolised to pyruvate, which can be converted to lactate and enter the systemic circulation, thereby providing substrate for oxidation and/or contributing to glycogen storage in liver and muscle (Fig. 1) (Tappy & Lê, 2010). The quantity of fructose directly contributing to DNL in the liver is low (Tappy & Lê, 2010). However, tracing the fate of fructose carbons alone (e.g. via carbon‐labelled fructose) does not necessarily provide full insight into DNL from all precursors. For example, in addition to serving as a precursor, fructose availability (directly or indirectly via hepatic glycogen) could stimulate the *process* of DNL and thus increase the conversion of precursors such as glucose, lactate and fructose to triglycerides. In this regard, the use of deuterium oxide to determine DNL (Pinnick *et al*. 2019) has advantages over labelled fructose.

**Figure 2:**
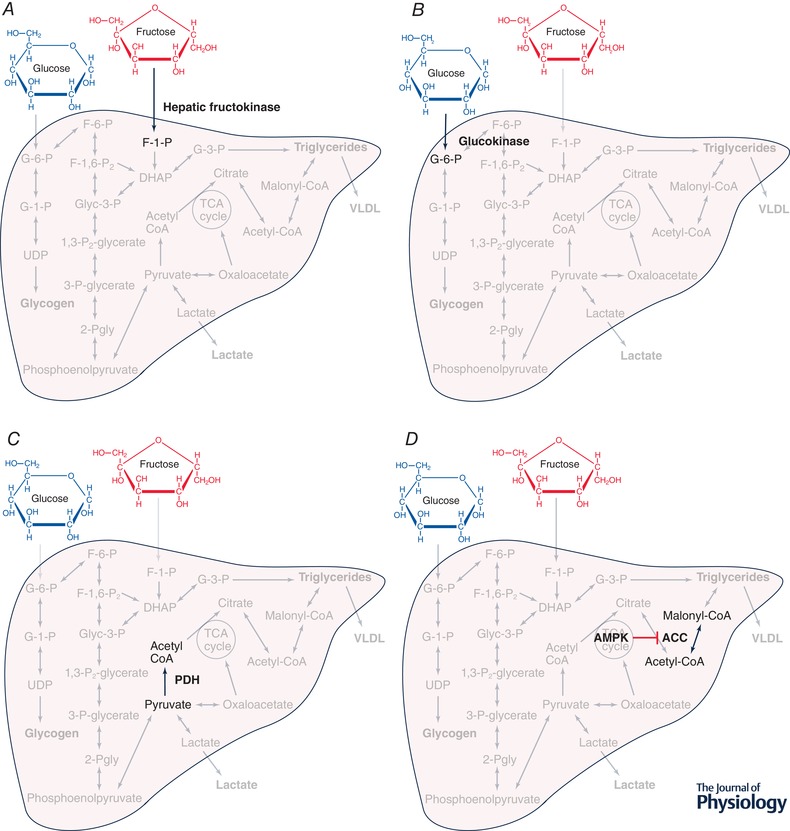
Highlighting some of the important pathways in hepatic carbohydrate handling and *de novo* lipogenesis *A*, fructose is converted into fructose‐1‐phosphate via enzyme hepatic fructokinase. *B*, glucose is converted into glucose‐6‐phosphate via enzyme glucokinase. *C*, pyruvate is converted into acetyl‐coenzyme A via enzyme pyruvate dehydrogenase. *D*, acetyl coenzyme A is converted into malonyl coenzyme A via enzyme acetyl coenzyme A carboxylase, an enzyme which is negatively regulated by AMP‐activated protein kinase. F‐1‐P, fructose‐1‐phosphate; 6‐G‐P, glucose‐6‐phosphate; Acetyl‐CoA, acetyl coenzyme A; PDH, pyruvate dehydrogenase; ACC, acetyl CoA carboxylase; AMPK, AMP‐activated protein kinase; Malonyl‐CoA, malonyl coenzyme A.

Acute co‐ingestion of fructose with glucose results in greater hepatic DNL than from glucose ingestion alone (Parks *et al*. 2008), and whilst fasting DNL is not upregulated following 10 weeks of fructose feeding in overweight humans (Stanhope *et al*. 2009), postprandial DNL is massively upregulated (>7‐fold) following overfeeding with fructose compared with glucose for 10 weeks (Stanhope *et al*. 2009). The regulation of DNL in the presence of fructose is complex, with many contributing processes (Fig. 1). Whereas hepatic glucose uptake is tightly regulated by a combination of hyperglycaemia, hyperinsulinaemia and delivery of glucose via the portal vein (McGuinness & Cherrington, 2003), hepatic fructose uptake occurs in an ‘unregulated’ manner, without negative feedback, driven by hepatic fructokinase with high affinity for fructose (Fig. 2 *A*) (Adelman *et al*. 1967). DNL can occur from glucose, fructose and lactate as precursors via multiple pathways (Sanders & Griffin, 2016): either via increased glycolytic production of acetyl coenzyme A (acetyl‐CoA), or via accumulation of dihydroxyacetone phosphate from glycolytic (fructose‐1,6‐bisphosphate) or directly from fructolytic (fructose‐1‐phosphate) intermediates (Fig. 1). The role of fructose in liver metabolism has been reviewed in more detail previously (McGuinness & Cherrington, 2003; Bizeau & Pagliassotti, 2005; Geidl‐Flueck & Gerber, 2017); however, to understand how fructose influences hepatic DNL, we will focus on the role of transcriptional and allosteric regulation, the role of energy sensing and AMP‐activated protein kinase (AMPK), and the interplay between β‐oxidation and lipogenesis.

### Transcriptional and allosteric regulation

Fructose interacts with various transcriptional and allosteric (enzymatic) processes along the pathways of glycogen synthesis, glycolysis and DNL within the liver. Transcriptional regulation translates into long‐term mechanisms of regulation while allosteric (enzymatic) processes will be responsible for acute/immediate mechanisms of regulation.

Sterol regulatory element binding proteins (SREBPs) are transcription factors with three isoforms (SREBP‐1a, ‐1c, ‐2) (Brown & Goldstein, 1997). The SREBP‐1c isoform activates transcription of numerous genes encoding lipogenic enzymes (Horton, 2002). Insulin stimulates transcription of the gene coding for SREBP‐1c and glucagon inhibits this transcription (Goldstein *et al*. 2006). SREBP‐1c is thought to be the primary mediator of insulin‐induced hepatic lipogenesis because hepatic SREBP‐1c transcription decreases in the liver of rats treated with streptozotocin (which ablates insulin secretion) and rises back to normal levels following insulin treatment (Shimomura *et al*. 1999). However, insulin‐independent hepatic SREBP‐1c activation can be achieved in rats by refeeding with glucose, sucrose and fructose following streptozotocin administration (Matsuzaka *et al*. 2004), suggesting that both nutrient availability and insulin concentrations play a role in SREBP‐1c‐stimulated hepatic lipogenesis. Fructose feeding in rats also stimulates peroxisome proliferator‐activated receptor γ coactivator‐1 β (PGC‐1β) (Nagai *et al*. 2009), which is a transcriptional coactivator of SREBP‐1c. With regards to SREBP‐1c, fructose may be of secondary importance to hepatic lipogenesis compared with the presence of high insulin concentrations, but clearly plays a role in this pathway.

Carbohydrate response element binding protein (ChREBP) is a transcription factor which is activated by carbohydrate feeding and is expressed in the liver of rats (Yamashita *et al*. 2001) and humans (Hurtado del Pozo *et al*. 2011), and which also targets numerous lipogenic enzymes (Ma *et al*. 2006). It has been hypothesised that fructose may activate hepatic ChREBP to a greater extent than glucose due to the unrestricted nature of hepatic fructose uptake (Ter Horst & Serlie, 2017). Interestingly, in ChREBP knockout mice, a high‐fructose diet does not lead to liver fat accumulation but instead accelerates fibrosis (Zhang *et al*. 2017b), suggesting that ChREBP activation is a necessary signal to allow fructose‐induced hepatic lipogenesis to occur.

Glucokinase is the enzyme which phosphorylates glucose to glucose‐6‐phosphate (Fig. 2 *B*) and therefore is responsible for facilitating hepatic glucose uptake. Glucokinase is tightly regulated by negative feedback loops, mainly via the glucokinase regulatory protein (GKRP) which binds to, and inhibits activity of, glucokinase (Van Schaftingen, 1989). Fructose‐6‐phosphate promotes GKRP to bind to glucokinase, inhibiting its activity, whereas fructose‐1‐phosphate has the opposite effect on GKRP, facilitating its dissociation from the glucokinase, which increases glucokinase activity (Vandercammen & Van Schaftingen, 1990; Vandercammen *et al*. 1992). Therefore, greater fructose‐1‐phosphate concentrations result in greater hepatic glucose uptake via glucokinase (Davies *et al*. 1990). This has been demonstrated *in vivo* in dogs, where small doses of fructose infused to the portal vein result in increased hepatic glucose uptake, hepatic glycogen synthesis, and hepatic glycolysis (Shiota *et al*. 2002). The extent to which hepatic fructokinase phosphorylates fructose to fructose‐1‐phosphate is not regulated and instead is driven primarily by the availability of fructose to the liver (Tappy & Lê, 2010). This provides mechanistic evidence for how hepatic fructose uptake potentiates hepatic glucose uptake, which supports evidence in humans that glucose–fructose co‐ingestion approximately doubles hepatic glycogen repletion rates compared with glucose ingestion alone (Gonzalez *et al*. 2017).

Pyruvate dehydrogenase (PDH) is a tightly regulated protein complex in mitochondria which catalyses the decarboxylation of pyruvate to acetyl‐CoA (Fig. 2 *C*) (Harris *et al*. 2002), which is an irreversible step in hepatic lipogenesis. PDH is activated by insulin and Ca^2+^ via activation of pyruvate dehydrogenase phosphatase (Harris *et al*. 2002). PDH is inactivated by acetyl‐CoA and NADH, via activation of pyruvate dehydrogenase kinase (Harris *et al*. 2002). In rats, prolonged fructose ingestion stimulates hepatic PDH activity and increases hepatic DNL compared with prolonged glucose ingestion. However, a direct role of PDH activity in hepatic lipogenesis is unclear, since knockout and pharmacological inhibition of hepatic pyruvate dehydrogenase kinases both suppress hepatic ChREBP‐mediated lipogenesis (Wu *et al*. 2018). Furthermore, the extent to which PDH activity is quantitatively important for human hepatic lipid metabolism is currently unclear.

Acetyl‐CoA carboxylase (ACC) is an enzyme responsible for catalysing acetyl‐CoA to malonyl coenzyme A (malonyl‐CoA) in the first ‘committed’ step to lipogenesis (Fig. 2 *D*) (Hardie, 1989). In rat hepatocytes, ACC is stimulated by both glucose and insulin (Katz & Ick, 1981) and is inhibited by phosphorylation with AMP‐activated protein kinase (AMPK) (Carling *et al*. 1987). In rodents, high‐fructose feeding upregulates ACC activity (Winder *et al*. 1975), and pharmacological inhibition of ACC can decrease hepatic DNL and hepatic steatosis (Goedeke *et al*. 2018). This is, however, at the expense of hypertriglyceridaemia (Goedeke *et al*. 2018). In human‐derived HEPG2 cells, addition of fructose to glucose does not further upregulate ACC expression (Hirahatake *et al*. 2011), so it is unclear in humans how an acute physiological dose of fructose directly influences hepatic ACC activity. Altered ACC activity from fructose intake may therefore require sustained increases in fructose intake and may therefore interact with energy sensing pathways as the energy and glycogen status of the hepatocytes are changed.

### Energy sensing and AMP‐activated protein kinase signalling

The AMP/ATP ratio within eukaryotes determines the principal energy status of the cell, and the major, most widely conserved indicator of this ratio is AMPK (Herzig & Shaw, 2018). AMPK is activated by metabolic stressors including nutrient deprivation, for example depriving hepatocytes or pancreatic β‐cells of glucose markedly upregulates AMPK activity (Salt *et al*. 1998; Zhang *et al*. 2013). It is now well established that AMPK is activated under conditions of low‐energy status and that this inhibits anabolic pathways and promotes catabolic pathways. As mentioned previously, this is mediated via the phosphorylation of ACC with an inhibitory effect (Fig. 2 *D*) (Carling *et al*. 1987). Refeeding rats with carbohydrate following fasting results in a reduction of hepatic AMPK activity whilst concurrently increasing hepatic ACC activity (Assifi *et al*. 2005), and AMPK activation via a gain‐of‐function mouse model has been shown to inhibit hepatic DNL without influencing lipid oxidation (Woods *et al*. 2017).

Fructose stimulates hepatic DNL from multiple pathways (Fig. 1), which is potentially because hepatic fructokinase is not negatively regulated by ATP levels (Adelman *et al*. 1967), so fructolysis occurs independent of cellular energy status. It has recently been shown that fructose‐1,6‐bisphosphate, an intermediate of fructose metabolism (Fig. 1), impairs activation of AMPK during glucose starvation (Zhang *et al*. 2017a), suggesting that the presence of fructose metabolites *per se* accelerates DNL by reducing AMPK activity and increasing ACC activity. Furthermore, the phosphorylation of fructose to fructose‐6‐phosphate results in conversion of ATP to ADP (Hallfrisch, 1990), and this results in uric acid production (Nakagawa *et al*. 2006). This has been shown to increase lipogenesis and the expression of lipogenic genes in hepatocytes (Lanaspa *et al*. 2012), providing an additional mechanism related to energy sensing by which fructose increases DNL.

In addition to being regulated by the energy status of the cell via the ATP:AMP ratio, AMPK activity is also regulated by glycogen concentrations, independent of AMP. Glycogen binds to the β‐subunit of AMPK and this inhibits AMPK activity (McBride *et al*. 2009), at least in skeletal muscle. For example, increasing skeletal muscle glycogen content of rodents by ∼3‐fold decreases basal AMPK‐α2 activity almost proportionally to glycogen concentration. Furthermore, whilst AMPK‐α2 activity is still responsive to stimulation by the AMP analogue, 5‐aminoimidazole‐4‐carboxamide ribonucleotide (AICAR), this stimulated response is drastically blunted in the presence of high *versus* low muscle glycogen concentrations (Wojtaszewski *et al*. 2002). Therefore, across a range of AMP concentrations, AMPK activity is modulated by glycogen concentrations. Assuming similar AMPK regulation occurs in the liver, then high glycogen concentrations may inhibit AMPK activity, thus alleviating the inhibitory phosphorylation of ACC, allowing greater DNL to occur.

### Interplay between β‐oxidation and lipogenesis

Net hepatic lipid production is the summation of either reduced β‐oxidation, increased lipogenesis, or a combination of both processes. Evidence discussed so far indicates that fructose upregulates lipogenic processes in the liver, but there is also evidence that β‐oxidation is reduced, thereby further contributing to net lipid production. Peroxisome proliferator‐activated receptor α (PPARα) is the master regulator of hepatic lipid metabolism and expression is induced by fasting (Kersten & Stienstra, 2017). In human muscle, glucose and insulin inhibit fat oxidation by reducing the rate of fatty acid entry to the mitochondria (Sidossis *et al*. 1996). The mechanism of inhibition in rat hepatocytes is an increase in cellular malonyl‐coenzyme A (McGarry *et al*. 1977). Fructose has been shown to inhibit β‐oxidation in rat and human hepatocytes (Rebollo *et al*. 2014), and chronic fructose ingestion in rats leads to an increase in hepatic lipogenic genes as well as a concurrent decrease in hepatic carnitine palmitoyltransferase 1 (CPT1) (Teofilovic *et al*. 2016). This suggests that fructose increases net hepatic lipid production from multiple mechanisms, not solely an increase in lipogenesis.

## A role for hepatic glycogen in regulating *de novo* lipogenesis?

Hepatic glycogen content reflects the balance between glycogen synthesis (via direct and indirect pathways), and glycogenolysis. As mentioned in the previous section, fructose markedly upregulates hepatic glycogen synthesis as well as upregulating hepatic DNL. Whilst this suggests that fructose has the potential to enhance the recovery of athletic performance (Fuchs *et al*. 2019), it also kindles the idea that hepatic glycogen content could be a key regulator of hepatic lipid metabolism.

**Figure 3:**
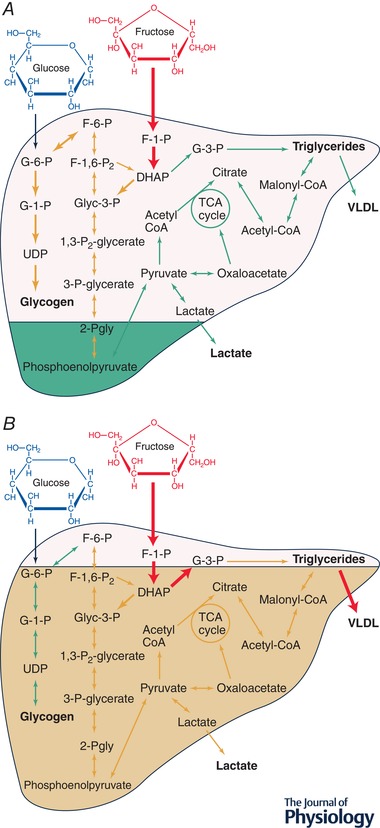
The role of glycogen status on hepatic *de novo* lipogenesis and VLDL export The traffic light system demonstrates flux through a given pathway, where red (thickest lines in black and white version) represents high flux, orange (medium thickness lines in black and white version) represents medium flux, and green (thinnest lines in black and white version) represents low flux. *A*, low hepatic glycogen status enables ingested fructose to stimulate hepatic glycogen synthesis, allowing flux through lipogenic pathways to remain low. *B*, high hepatic glycogen status determines that ingested fructose is shunted away from glycogen synthesis and towards lipogenic pathways, which leads to greater VLDL export. G‐6‐P, glucose‐6‐phosphate; G‐1‐P, glucose‐1‐phosphate; UDP, uridine diphosphate glucose; F‐1‐P, fructose‐1‐phosphate; F‐6‐P, fructose‐6‐phosphate; F‐1,6‐P_2_, fructose‐1,6‐bisphosphate; Glyc‐3‐P, glyceraldehyde‐3‐phosphate; 1,3‐P_2_‐glycerate, 1,3‐bisphosphoglycerate; G‐3‐P, glycerol‐3‐phosphate; 3‐Pgly, 3‐phosphoglycerate; 2‐Pgly, 2‐phosphoglycerate; Acetyl‐CoA, acetyl coenzyme A; Malonyl‐CoA, malonyl coenzyme A; DHAP, dihydroxyacetone phosphate; VLDL, very‐low‐density lipoprotein.

Mice with the gene coding for hepatic glycogen synthase (*Gys2*) knocked down display markedly increased hepatic DNL, which leads to hepatic insulin resistance and steatosis (Irimia *et al*. 2017), which suggests that the inability to synthesise hepatic glycogen is a driver of hepatic DNL. In the alternative scenario, in glycogen storage disease type 1a, where glycogen stores are saturated (Cori & Cori, 1952), hepatic DNL is also markedly upregulated (Bandsma *et al*. 2008). Due to the stimulatory effects of fructose on hepatic glycogen synthesis discussed in the previous section, liver glycogen stores may become ‘saturated’ in scenarios where fructose is ingested in large quantities, when glycogen is not utilised at a high rate (i.e. during sedentary conditions), or a combination of these two conditions, and thus, excess carbohydrate is converted into lipid (Fig. 3).

In scenarios where hepatic glycogen stores are already ‘full’, hepatic glucose and fructose uptake may be shunted more towards DNL, whereas when hepatic glycogen stores are low, glucose and fructose are potentially shifted more towards glycogen synthesis rather than DNL. This is supported by evidence that hepatic glycogen stores undergo autoregulation, whereby low glycogen concentrations stimulate glycogen synthesis (Fleig *et al*. 1987) and inhibit glycogenolysis (Roden *et al*. 2001). Furthermore, overriding this autoregulation by overexpressing hepatic glucokinase to accelerate hepatic glugose uptake in an unregulated fashion results in markedly increased hepatic glycogen stores, but at the expense of hypertriglyceridaemia (potentially from DNL) (O'Doherty *et al*. 1999). However, if glycogen synthesis is stimulated directly – by overexpressing hepatic protein targeting to glycogen – there are no changes in triglyceridaemia (O'Doherty *et al*. 2000). This suggests that the mechanism binding glycogen stores to fructose‐induced increases in hepatic DNL could be the metabolic fate of hepatic carbohydrate uptake: directed more to glycogen when glycogen stores are low.

Assuming the hepatic glycogen hypothesis is correct, the ingestion of fructose when hepatic glycogen stores are already saturated would stimulate lipogenesis to a greater extent than when hepatic glycogen stores are low. If glycogen is being utilised at a rate high enough to overcome net glycogen synthesis, despite fructose intake, then theoretically this should reduce hepatic DNL and VLDL output. Whilst there are no data in humans, in rats daily exercise can mediate the initial increase in hepatic lipogenic gene expression after 3 days of high‐fructose feeding (Winder *et al*. 1975). It will be intriguing to explore whether the capacity to store liver glycogen correlates with the lipogenic response to fructose ingestion. With this in mind, from a practical standpoint, to mitigate negative metabolic effects of fructose intake via modulating hepatic glycogen stores, perhaps the advice should be to ‘deplete before you eat’? In support of this, a single bout of exercise, which lowers glycogen content in liver and skeletal muscle, has been shown to potently downregulate postprandial hepatic DNL and liver triglyceride storage in humans with insulin resistance (Rabøl *et al*. 2011).

## Considering nutrient–physical activity interactions

Physiology is fundamentally the study of life, and nutrients are essential for life to be maintained, but it is an oversimplification to view the intake of any nutrient as a sole determinant of one of the many processes of life. Instead, physiology is dictated by interactions between nutrients and many other stimuli, and it is this integrated view that we should strive towards in order to understand the physiological effects of nutritional habits. In a world where obesity and associated metabolic complications are rising (NCD Risk Factor Collaboration, 2016), understanding the role of nutrients in energy balance and metabolism is more pertinent than ever. Considering evidence relevant to fructose, diets high in sucrose have been correlated with higher energy intake (Johnson *et al*. 2009), and adding sucrose to or removing it from the diet leads to a modest increase or decrease in weight, respectively, over time (Te Morenga *et al*. 2013). However, sucrose is unlikely to have a role solely on energy intake independent from any other component of energy balance, and instead could influence other facets of energy intake and/or energy expenditure, including physical activity energy expenditure.

At the extremes of physiology, ‘simple carbohydrate’ (sugars) intake of ∼460 g (∼1720 kilocalories), and total energy intake of ∼5800 kilocalories, per day are achieved in Tour de France cyclists (Saris *et al*. 1989), yet they do not develop metabolic disorders or hepatic steatosis. Similarly, some hunter gatherer populations are reported to consume as much as 50% of energy intake from honey, but also appear to have very low prevalence of metabolic disease (Pontzer *et al*. 2018). In non‐exercising humans, similar nutrient intake results in metabolic detriment and high rates of net DNL within days (Acheson *et al*. 1988). Thus, these two similarly extreme nutrient intakes exert quite contrasting physiological effects depending on the physiological state of the individual, and hepatic glycogen content could be a key factor that dictates these responses.

The possibility that hepatic glycogen stores are important for mediating fructose‐driven hepatic DNL is a good example of why nutrient–physical activity interactions are important to consider. Acute endurance exercise increases hepatic glycogenolysis, and is largely dictated by the exercise intensity – especially in untrained individuals – (Gonzalez *et al*. 2016). However, even in untrained individuals, it would take ∼2 h exercising at 80% ${\dot{V}}_{O_{2}\max}$ for liver glycogen to be depleted to very low levels (>70%) (Gonzalez *et al*. 2016). Whilst intensity is clearly important for hepatic glycogen stores, this suggests that total volume and patterns of physical activity are also important considerations. Where possible, researchers should strive to understand the effects of fructose ingestion (or any other nutrient) in the context of mediating factors. Measuring physical activity energy expenditure, type, timing and intensity are all useful towards understanding the physiological effects of nutrient ingestion.

## Conclusions

Dietary fructose plays an important role in hepatic glycogen and lipid metabolism, with potential consequences for metabolic health. Ingestion of fructose increases rates of hepatic *de novo* lipogenesis and, in the context of a positive energy balance, can lead to greater VLDL export and hypertriglyceridaemia. An inability to further synthesise glycogen upregulates hepatic DNL and, considering that fructose ingestion increases both DNL and hepatic glycogen synthesis, glycogen stores may play a key role in determining the metabolic responses to fructose ingestion. The corollary is that negative metabolic effects of fructose intake are most likely to manifest when hepatic glycogen stores are saturated. This hypothesis provides a rationale for striving to consider nutrient–physical activity interactions in physiology research, and to target turnover and/or utilisation of hepatic glycogen stores to improve metabolic health. Future research should strive to take an integrated approach towards understanding physiological responses to nutrients.

## Additional information

### Competing interests

None declared.

### Author contributions

All authors have approved the final version of the manuscript and agree to be accountable for all aspects of the work. All persons designated as authors qualify for authorship, and all those who qualify for authorship are listed.

### Funding

The present work was not supported by any specific funding source. A.H. is completing a PhD studentship with financial support from The Rank Prize Funds and the University of Bath. Additional funding for consumables is provided by Kenniscentrum Suiker & Voeding. F.K. has received funding from the Medical Research Council, Diabetes UK and The British Heart Foundation in the past. She is currently funded by the Medical Research Council (MR/P002927/1). J.T.G. has received funding from The European Society for Clinical Nutrition and Metabolism (ESPEN), The Rank Prize Funds, Kenniscentrum Suiker & Voeding, Arla Foods Ingredients, the Medical Research Council, and the Biotechnology and Biological Sciences Research Council. J.T.G. has also acted as a consultant to PepsiCo and Lucozade Ribena Suntory.
